# Cancer Prevention Europe

**DOI:** 10.1002/1878-0261.12455

**Published:** 2019-02-13

**Authors:** Christopher P. Wild, Carolina Espina, Linda Bauld, Bernardo Bonanni, Hermann Brenner, Karen Brown, Joakim Dillner, David Forman, Ellen Kampman, Mef Nilbert, Karen Steindorf, Hans Storm, Paolo Vineis, Michael Baumann, Joachim Schüz

**Affiliations:** ^1^ International Agency for Research on Cancer (IARC/WHO) Lyon France; ^2^ Usher Institute College of Medicine and Veterinary Medicine University of Edinburgh UK; ^3^ Cancer Research UK London UK; ^4^ European Institute of Oncology Milano Italy; ^5^ German Cancer Research Center (DKFZ) Heidelberg Germany; ^6^ UK Therapeutic Cancer Prevention Network Leicester Cancer Research Centre University of Leicester UK; ^7^ Karolinska University Laboratory Karolinska University Hospital Stockholm Sweden; ^8^ Division of Human Nutrition and Health Wageningen University The Netherlands; ^9^ Danish Cancer Society Copenhagen Denmark; ^10^ Department of Epidemiology and Biostatistics School of Public Health Imperial College London UK

**Keywords:** cancer, Cancer Prevention Europe, Europe

## Abstract

The case for cancer prevention in Europe is the same as for all other parts of the world. The number of cancers is increasing, driven by demographic change and evolution in the exposure to risk factors, while the cost of treating patients is likewise spiralling. Estimations suggest that around 40% of cancers in Europe could be prevented if current understanding of risk and protective factors was translated into effective primary prevention, with further reductions in cancer incidence and mortality by screening, other approaches to early detection, and potentially medical prevention. However, the infrastructure for cancer prevention tends to be fragmented between and within different countries in Europe. This lack of a coordinated approach recently led to the foundation of Cancer Prevention Europe (Forman *et al*., 2018), a collaborative network with the main aims of strengthening cancer prevention in Europe by increasing awareness of the needs, the associated required resources and reducing inequalities in access to cancer prevention across Europe. This article showcases the need for strengthening cancer prevention and introduces the objectives of Cancer Prevention Europe and its foreseen future role in reducing the European cancer burden.

**Abbreviations**
BBMRI: Biobanking and BioMolecular resources Research and Infrastructure
EU: European Union
HPV: human papillomavirus
NCDs: noncommunicable diseases
WHO: World Health Organization

## 1 The case for prevention

**Figure 1:**
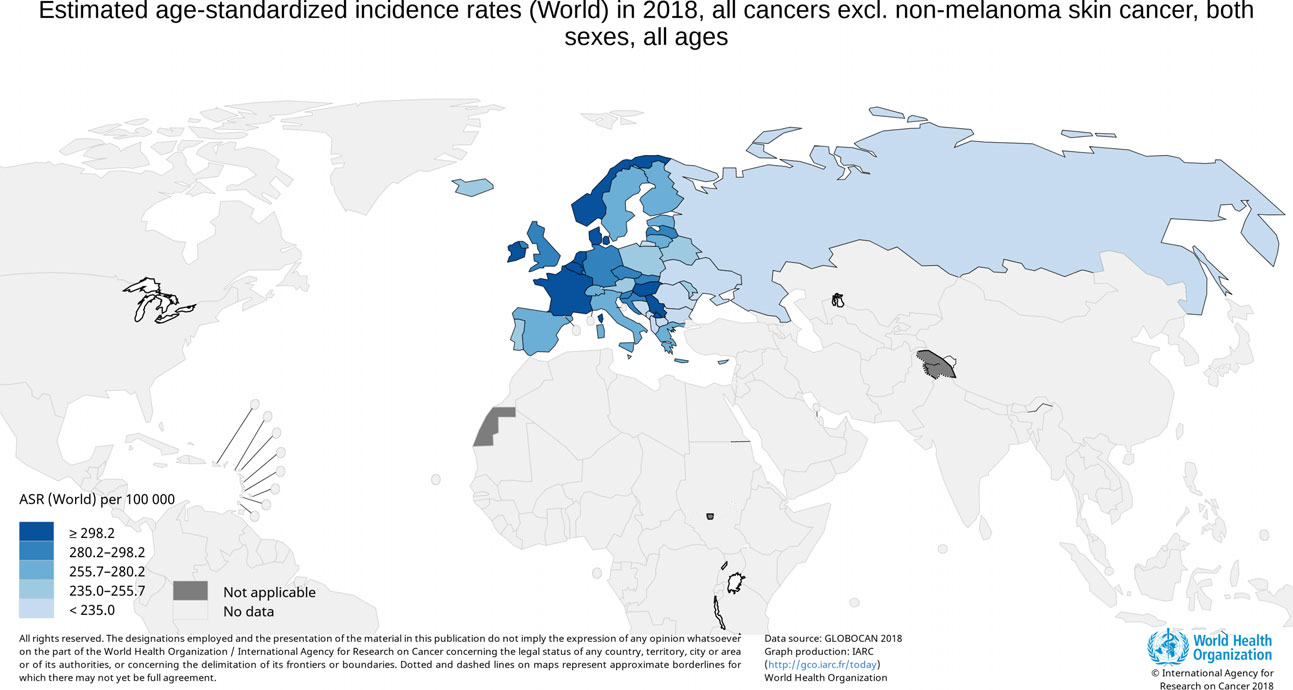
Age‐standardized cancer incidence rate in European countries (both sexes combined, excluding nonmelanoma skin cancer, using World Standard Population).

The case for cancer prevention in Europe, at a fundamental level, is the same as for all other parts of the world. The number of cancers is increasing, driven by demographic change and evolution in the exposure to risk factors, while the cost of treating patients is likewise spiralling. The most recent report on 25 cancers in the 40 countries of Europe estimated 3.91 million new cases (excluding nonmelanoma skin cancer) and 1.93 million deaths in 2018 (Ferlay *et al*., 2018). This corresponds to an age‐standardized cancer incidence rate of 374 cases per 100 000 population (European age standard), with some variation across the countries (Fig. 1). In the European Union (EU)‐28, the estimated number of new cases of cancer was ~ 1.6 million in males and 1.4 million in females, with 790 000 men and 620 000 women dying from the disease. The number of cancers on the continent is projected to increase to 4.75 million cases and 2.55 million deaths in 2040 primarily as a result of population ageing and growth (GCO 2018). This represents an overall increase in mortality of 32%, that is an additional 620 000 people dying each year.

In Europe, other noncommunicable diseases (NCDs), including cardiovascular disease, diabetes and chronic respiratory illnesses, also place a heavy burden on health systems, but here progress in treating or controlling the diseases in their early phases has been more effective. The complexity and diversity of cancer, occurring as it does in different organs and cell types with associated intratumour heterogeneity, implies the need for a multitude of tests for early detection coupled with treatments tailored to specific types of cancers. This is quite different in scope to other NCDs where, for example, progress has been made in controlling blood pressure or cholesterol through the development of widely applicable drugs, such as antihypertensives and statins. The result is that cancer is now the leading cause of premature death (defined as death below the age of 70 years) in 28 of the 40 countries of Europe and is the second most common in the remaining countries (Ferlay *et al*., 2018). Furthermore, while there is good news in terms of improvement in cancer survival in Europe, cancer survivorship also entails long‐term follow‐up and care with the attendant demands on health services (Allemani *et al*., 2018).

In contrast to the dominance of cancer in terms of disease burden in Europe and the high proportion of cancers attributable to modifiable factors, the majority of cancer research investment continues to be made in basic science and clinical translational research with the focus on the development of new therapies or improving treatment. In addition, investment in primary prevention has often been neglected partly because the results are difficult to recognize in individuals and its impact may take several decades to emerge. For example, in data provided by the International Cancer Research Partnership for the United Kingdom (UK), France and the Netherlands (for 2014 and 2015, the last years with complete data), 57% of funding from government and nongovernment organization sectors was assigned to therapy‐oriented biology and drug development with 7% to prevention and 13% each to aetiology and to early detection, diagnosis and prognosis (ICRP 2018). Private sector funding of cancer research has little incentive to invest in prevention, besides research related to vaccine and early detection technology development, and thus, the balance is further skewed if an analysis of all funding sources is conducted. Exciting developments in precision oncology drugs and immunotherapy promise a step‐change improvement in cancer survival, but come at high cost and by definition benefit a few subsets of patients.

It is not alarmist to conclude that the status quo in relation to cancer control measures threatens the sustainability of healthcare provision in Europe. The economics of a primary focus on cancer treatment do not make for cost‐effective cancer control policies unless aligned to public health strategies for prevention.

There are evidence‐based and cost‐effective preventive interventions available for cancer, based on prior research into aetiology. In addition, primary prevention offers the most advantageous approach to reducing cancer and other NCDs by reducing common risk factors and therefore producing important co‐benefits for health (Espina *et al*., 2013). Primary prevention coupled with secondary prevention through early detection of premalignancy can avoid not only medical costs, but also the considerable physical, social and psychological comorbidities and suffering associated with most cancer treatments. Tertiary prevention of cancer recurrence among survivors adds further weight to a balanced approach to prevention and treatment. Indeed from a health, social and economic viewpoint, a more systematic and structured approach to cancer prevention in Europe is a logical necessity.

Any strategic approach to cancer prevention in Europe needs to recognize heterogeneity across the continent, both in the pattern of cancers and the stage of implementation of the available preventive interventions. In Bulgaria, Romania and some Baltic States, for example, the prevalence of persistent human papillomavirus (HPV) infection is rising in the absence of HPV vaccination, and as a consequence of this and of the lack of effective screening, cervical cancer incidence rates are on the increase (Arbyn *et al*., 2017). In France, the HPV vaccination rate among young girls is only around 25%. Europe has the highest smoking rates of any World Health Organization (WHO) region, but this masks considerable differences (EURO 2017). While countries like Sweden, Iceland, Ireland, Norway and the UK have achieved significant reductions in smoking in recent years, other countries like Hungary and Cyprus have seen little change. Alcohol consumption is the second most common cause of cancer in France (Shield *et al*., 2017), after smoking, with this risk factor having been under‐emphasized as a priority for cancer prevention, as has avoidance of excess exposure to sunlight. The most recent report on implementation of cancer screening programmes for cervix, breast and colorectal cancers shows a general improvement in the EU, but nevertheless reveals marked differences among countries (Ponti *et al*., 2017).

Recent detailed estimates in France, the UK and Germany suggest that around 40% of cancers in Europe could be prevented if current understanding of established risk and protective factors was translated into effective primary prevention (Behrens *et al*., 2018; Brown *et al*., 2018; Gredner *et al*., 2018; Mons *et al*., 2018; Soerjomataram *et al*., 2018). Cancer screening and other approaches to early detection of premalignant lesions or surveillance among very high‐risk groups can also contribute to reduce cancer incidence and mortality. Interventions such as physical activity among breast cancer survivors offer exciting opportunities to improve prognosis and quality of life among cancer survivors (Friedenreich *et al*., 2017). In due course, additional benefits may come from medical prevention among cancer survivors or through surveillance of high‐risk individuals or groups in the general population (Cuzick, 2017).

Successful cancer prevention is not a trivial challenge. It requires considerable commitment to implementation at national level through strategies that reach all segments of society. Solutions cannot be aimed only at individuals (as characterized by the European Code against Cancer (Schuz *et al*., 2015)) but must be supported by legislative and regulatory measures. Some exposures, notably reduction in exposure to air pollution, require international agreements in order to be truly effective. A cautionary note is merited in some areas of prevention where ‘more is less’, either because approaches being implemented are not evidence‐based or because the magnitude of any effect would be insignificant. An example is the over‐diagnosis and over‐treatment of some cancers, for example, small papillary thyroid cancers (Vaccarella *et al*., 2016).

Notwithstanding the challenges in implementing preventive interventions, the prize is of great value and complementary to that of treating and caring for cancer patients more effectively. Reducing the number of patients developing cancer should result in greater resources being available to treat those patients with the most effective therapies available.

## 2 The need for strengthening cancer prevention in Europe

Cancer prevention has a broad scope. As mentioned above, the field encapsulates surveillance and descriptive data (e.g. incidence, mortality, survival and prevalence; economic analyses including cost‐effectiveness; prevalence of exposure to risk factors) as well as the areas of primary, secondary and tertiary prevention. Prevention may be aimed at the whole population, for example as with antismoking legislation, or at specific high‐risk subgroups, for example, surveillance colonoscopy in patients previously diagnosed with polyps, with aspirin being under consideration for different at‐risk groups.

The broad scope of prevention is naturally matched by a broad scope of practitioners. Indeed, the full range of prevention activities relies on an interdisciplinary approach that encompasses epidemiology, cancer registries, basic and applied laboratory sciences, public health, general practice, clinical science, health services, health psychology, the social sciences and implementation science among other disciplines. The contribution of social sciences, humanities and anthropology is particularly needed. It is now perceived that the traditional health promotion/health education paradigm based on individual advice (e.g. from physicians or nurses), though laudable, is not sufficient and tends to create social disparities in terms of efficacy. Social sciences, and particularly anthropology, help to embed behavioural changes in cultural contexts. This is particularly true, for example, of obesity, which is not equally perceived in all social strata and cultural subgroups. Indeed, all cancer control initiatives should undergo a thorough and ongoing evaluation as to whether they diminish or exacerbate social inequalities within and between countries (Vaccarella *et al*., 2018).

The broad scope of disciplines brings with it a broad scope of institutions and professional organizations. Perhaps partially as a result of this situation, at institutional or even national level the infrastructure for cancer prevention tends to be fragmented. There are few exemplars of ‘prevention centres’ analogous to primary, secondary or tertiary care centres. Likewise, there are few centres of research excellence in prevention, unlike the many world class cancer treatment centres in Europe. International collaborative consortia (e.g. the European Organisation for Research and Treatment of Cancer) are the norm in the employment of clinical trials in development of new treatments, whereas the absence of analogous structures is one factor which inhibits the development of world class prevention research.

Most often there is a separation between centres of expertise in cancer prevention and in cancer treatment, reflected in differences in organizational responsibility, perception and culture. Health care is usually the political responsibility of Government (Ministry of Health or equivalent, and their respective health authorities), performed by healthcare professionals and undertaken in hospitals and primary care centres. These parameters of responsibility, expertise and location are considerably more complicated for prevention. Many of the above‐mentioned disciplines required for cancer prevention are to be found, for example, in institutes of public health, universities, charities, and health and non‐health‐related government departments or cancer centres. Linkages in the context of cross‐sectorial initiatives or strategies such as ‘Health in All Policies’ would make good sense in cancer prevention (Espina *et al*., 2013).

Successful coordination of cancer prevention in Europe requires long‐term vision, a dedicated research agenda, and strategically targeted funding. It also requires a sustainable infrastructure and cooperation between countries and programmes to fill gaps in the evidence base for prevention, to avoid common pitfalls in implementation and to share capacity for research training and quality improvement. Comprehensive Cancer Centres are in an excellent position to offer a pan‐European cancer research infrastructure, linking treatment and prevention with research and education, and thus connecting research with the healthcare systems (Celis and Pavalkis, 2017). For all these reasons, the initiative was taken within the FP7 Eurocan Platform project to create a network for strengthening cancer prevention in Europe, called ‘Cancer Prevention Europe’. Close collaboration between Cancer Core Europe and Cancer Prevention Europe, involving other organizations and stakeholders active in cancer prevention and treatment, will ensure that developments in understanding the causes of cancer will translate both into clinical and population‐based innovations and practices, addressing the whole cancer continuum in partnership.

## 3 The objectives of Cancer Prevention Europe

Cancer Prevention Europe originated in a general and collective recognition that cancer prevention in Europe is fragmented and lacks an overall strategy. In addition, the requirement for a more integrated approach in conjunction with related innovations in the area of cancer treatment was clear: the formation of Cancer Core Europe (Celis and Pavalkis, 2017) offered an opportunity in this respect.

Cancer Prevention Europe was created, therefore, initially as a consortium of a number of leading European research institutions (Cancer Research UK, London, UK; Danish Cancer Society, Copenhagen, Denmark; European Institute of Oncology, Milan, Italy; German Cancer Research Centre, Heidelberg, Germany; Imperial College London, London, UK; Karolinska Institute, Stockholm, Sweden; UK Therapeutic Cancer Prevention Network, Leicester, UK; World Cancer Research Fund International, London, UK/Wereld Kanker Onderzoek Fonds, Amsterdam, the Netherlands) committed to prioritizing cancer prevention. Each consortium member made a financial contribution to fund a small secretariat at IARC and to initiate specific strategic areas of collaborative research. A full description of the manifesto of the consortium has recently been published (Forman *et al*., 2018).

In brief, a number of objectives have emerged from this first phase in the development of the Cancer Prevention Europe initiative:

- To provide an infrastructure for coordinated cancer prevention research at the European level which is sustainable and open to expansion with new members over time;
- To communicate and disseminate to policymakers the opportunities and benefits of available preventive interventions;
- To formulate the scope of prevention research and to advocate for increased investment in this area;
- To drive innovative interdisciplinary research, including the opportunities afforded by advances in understanding cancer aetiology;
- To bridge the identification of risk factors through to the development and implementation of preventive interventions;
- To enable the translation of research on preventive interventions into effective cancer policy;
- To provide a platform for advocacy for cancer prevention among a wide set of stakeholder engagement, including citizens and patients.

The development of an alliance of organizations focused on cancer prevention also promises to provide a focal point for development of professional training and career development in an area where no simple career pathway is evident. This initiative should consider the provision of dedicated academic courses and qualifications in the area of cancer prevention, with teaching provided from among the different disciplines implicated.

There are a number of challenges facing Cancer Prevention Europe. First, the consortium needs to identify a mechanism within the European funding tools to obtain the required financing to fulfil its objectives. One option is the new ‘mission‐orientated’ research agenda, but this is not the only mechanism that can be envisaged. For now, the commitment is high among the founder members of the consortium, but accessible resources remain limited. Second, Cancer Prevention Europe needs to encompass innovative research and collaboration across the whole of Europe, including the specific challenges of inequalities both between and within countries. The consortium is thus seeking mechanisms to broaden participation and achieve this critical mass without diluting commitment and quality. Third, Cancer Prevention Europe recognizes the importance of an integrated approach that encompasses prevention and treatment in cancer research and cancer control: this requires a European‐level vision that carries all of these areas forward to deliver sustainable cancer services.

## 4 The next steps for Cancer Prevention Europe

The ambition of Cancer Prevention Europe is to transform the current research landscape through this new interdisciplinary consortium of institutes and organizations. The consortium aims to conduct innovative world class research capable of translation into effective cancer prevention guidelines and policies at national and international level. Cancer Prevention Europe offers an integrated infrastructure capable of delivering such high‐quality research in a collaborative, interdisciplinary manner.

The innovative science behind cancer prevention offers an opportunity to add value to a number of prior investments at the level of the European Commission, including large collaborative research studies, for example on the exposome (e.g. Exposomics, HELIX) and research infrastructure investments, including biobanks (Biobanking and BioMolecular resources Research and Infrastructure ‐ European Research Infrastructure Consortium) and large population‐based cohort studies of chronic diseases (e.g. Biobanking and BioMolecular resources Research and Infrastructure ‐ Large Prospective Cohorts), which all provide platforms for research on cancer prevention.

Inherent to the philosophy of Cancer Prevention Europe is the sharing of resources (including existing research platforms, biospecimen repositories and cohorts); the sharing of data (enabling multicentre, trans‐national research projects); and the sharing of information (through the creation of a central repository of information pertinent to cancer prevention). Suitable and acceptable legal frameworks would be established within Cancer Prevention Europe to permit information exchange, to monitor regulations and to highlight potential and actual barriers to progress through implemented legislation.

The new mission‐oriented approach to European research investment provides one major opportunity to enhance cancer prevention and better align the investments in research with the needs of Member States in relation to cancer control, thus optimizing benefits for all European citizens. This approach aligns well with the recent WHO European Health Report 2018 (EURO 2018) and the World Health Assembly 2017 resolution on cancer prevention and control (WHO 2017).

## Author contributions

All authors contributed to the writing of this review article.

## Conflict of interest

The authors declare no conflict of interest.
